# *Porphyromonas gingivalis* exacerbates ulcerative colitis via *Porphyromonas gingivalis* peptidylarginine deiminase

**DOI:** 10.1038/s41368-021-00136-2

**Published:** 2021-09-30

**Authors:** Xida Zhao, Jingbo Liu, Chong Zhang, Ning Yu, Ze Lu, Shuwei Zhang, Yuchao Li, Qian Li, Junchao Liu, Dongjuan Liu, Yaping Pan

**Affiliations:** 1 Department of Periodontology, School and Hospital of Stomatology, China Medical University, Liaoning Provincial Key Laboratory of Oral Diseases, Shenyang, China; 2 Center for Implant Dentistry, School and Hospital of Stomatology, China Medical University, Liaoning Provincial Key Laboratory of Oral Disease, Shenyang, China; 3 The Forsyth Institute, Cambridge, MA USA; 4 Department of Emergency and Oral Medicine, School and Hospital of Stomatology, China Medical University, Liaoning Provincial Key Laboratory of Oral Diseases, Shenyang, China

**Keywords:** Oral diseases, Diseases

## Abstract

Ulcerative Colitis (UC) has been reported to be related to *Porphyromonas gingivalis* (*P. gingivalis*). *Porphyromonas gingivalis* peptidylarginine deiminase (PPAD), a virulence factor released by *P. gingivalis*, is known to induce inflammatory responses. To explore the pathological relationships between PPAD and UC, we used homologous recombination technology to construct a *P. gingivalis* strain in which the PPAD gene was deleted (Δ*ppad*) and a Δ*ppad* strain in which the PPAD gene was restored (comΔ*ppad*). C57BL/6 mice were orally gavaged with saline, *P. gingivalis*, Δ*ppad,* or comΔ*ppad* twice a week for the entire 40 days (days 0−40), and then, UC was induced by dextran sodium sulfate (DSS) solution for 10 days (days 31−40). *P. gingivalis* and comΔ*ppad* exacerbated DDS-induced colitis, which was determined by assessing the parameters of colon length, disease activity index, and histological activity index, but Δ*ppad* failed to exacerbate DDS-induced colitis. Flow cytometry and ELISA revealed that compared with Δ*ppad*, *P. gingivalis*, and comΔ*ppad* increased T helper 17 (Th17) cell numbers and interleukin (IL)-17 production but decreased regulatory T cells (Tregs) numbers and IL-10 production in the spleens of mice with UC. We also cocultured *P. gingivalis*, Δ*ppad*, or comΔ*ppad* with T lymphocytes in vitro and found that *P. gingivalis* and comΔ*ppad* significantly increased Th17 cell numbers and decreased Treg cell numbers. Immunofluorescence staining of colon tissue paraffin sections also confirmed these results. The results suggested that *P. gingivalis* exacerbated the severity of UC in part via PPAD.

## Introduction

Periodontitis, the most common oral infectious disease caused by bacterial biofilms, results in the loss of periodontal attachment and alveolar bone, and it is a major etiology of tooth loss in adults^1^. Increasing evidence suggests that periodontitis is a risk factor for many systemic diseases, for instance, intestinal diseases^2,3^. Inflammatory bowel disease (IBD) is an idiopathic relapsing and remitting intestinal inflammatory disease, including ulcerative colitis (UC) and Crohn’s disease (CD). The etiology and pathogenesis of UC are the result of complex interactions among environmental factors, intestinal microorganisms, genetic susceptibility, and immune factors, among which immunomodulatory disorder is the key factor. The activation of intestinal mucosal immune responses is the immediate cause of the occurrence and development of UC and affects its prognosis^4^. Studies have demonstrated that UC patients possess a significantly higher incidence of periodontitis, deeper periodontal pocket depth, and more tooth loss than healthy controls^5,6^. Furthermore, oral bacteria derived from periodontitis may translocate to the intestine, and together with the intestinal microbiome, cause intestinal epithelial cell barrier dysfunction and amplify intestinal inflammation^7^. Kitamoto et al. found that periodontitis contributes to the progression of colitis via a dual microbiome and immune mechanism. After reaching a certain level, oral microbiota pathogens in saliva will colonize the intestine. In addition, the excessive oral bacteria can induce the abnormal immune responses by migratory T helper 17 (Th17) cells^8^. Alterations in the structure of the small intestine, including epithelial stratification, altered villous height, and neutrophil infiltration, are observed in rats with ligature-induced periodontitis^9^. Supplementation with intestinal probiotics may alleviate these defects^10^. Oral infection with *Porphyromonas gingivalis* impairs colonic motor functions, suggesting that periodontal pathogens can be defined as regulators of the host response in IBD^11,12^. Proinflammatory cytokines produced and activated locally in the oral cavity might enter the bloodstream and circulate to the intestines, thus impacting IBD^13,14^. This evidence indicates that periodontitis may be closely related to colitis through a substantially altered microbiome.

*P. gingivalis* is an anaerobic bacterium and a main periodontal pathogen that resides in periodontitis lesions^15^. *P. gingivalis* expresses many virulence factors, including proteases, endotoxins, organic acids, and others that directly attack gingival tissues^1,16^. These virulence factors also allow *P. gingivalis* to induce abnormal immune responses in the host and lead to an ecological imbalance between the host and microorganisms^17–19^. After the initial bacterial insult, the host’s immune/inflammatory responses, partially driven by T lymphocytes, play a great role in the breakdown of periodontal tissues^20,21^. Increased Th17 cell proportions and interleukin (IL)-17 levels were observed in local gingival tissues from patients with periodontitis compared with those from healthy controls^22,23^, and these changes were reflected in the systemic circulation in serum^24,25^. Regulatory T cells (Tregs) are strongly associated with disease progression and reduced immune-inflammatory responses and function by suppressing the proliferation and proinflammatory cytokine production of Th1 and Th17 cells^21^. Increasing the numbers of Tregs have been used to regulate the imbalance of Th17/Tregs and successfully reduce *P. gingivalis*-induced alveolar bone loss^26,27^. Th17 cells and Tregs also play crucially important roles during the advancement of DSS-induced colitis^28,29^. Th17 cells, through an IL-17-dependent mechanism, stimulate the activation of infiltrating inflammatory cells and induce diffuse hemorrhage of the colonic mucosa, accelerating crypt abscess formation and UC exacerbation^30^. Because they promote the production of the anti-inflammatory cytokines TGF-β and IL-10 or limit the amplification of proinflammatory Th17 cells to suppress ongoing colitis, Tregs are regarded as the primary immunosuppressive cells in UC^30^.

Peptidylarginine deiminase (PAD) is a protein-modifying enzyme and a critical virulence factor of *P. gingivalis*, and it was first described in 1981^31^. PAD participates in many vital physiological processes, including cellular differentiation, immune responses, and gene transcription^32,33^. PAD can modify and transform the host proteins by catalyzing and converting peptidyl-arginine to peptidyl-citrulline—a procedure known as citrullination^34^. Protein citrullination can elicit an abnormal autoimmune response by changing the immunological activity of chemokines and altering the structure and function of proteins^35,36^. Disruptions of normal PAD activities lead to abnormal citrullination, resulting in multiple inflammatory diseases, such as rheumatoid arthritis, Alzheimer’s disease, and UC^37,38^. Citrullination stimulates the activation of neutrophils and macrophages in the initial stages of innate immune responses and promotes the progression of intestinal inflammation^39^. The role of citrullination in IBD was first reported in 2006, and the levels of citrullinated peptides in colonic biopsies from IBD patients were higher than those from the control subjects^40^. Immunohistochemistry showed that PAD staining was strong in the intestinal lamina propria cells of mice with DSS-induced acute colitis and patients with UC, confirming that citrullination can promote inflammation^28^. *Porphyromonas gingivalis* peptidylarginine deiminase (PPAD) is synthesized and released by *P. gingivalis* via membrane vesicles^41,42^, and can accelerate protein citrullination in gingival tissues. Whether the PAD secreted by *P. gingivalis* contributes to UC has not been reported.

In this study, we explored the significant effect of *P. gingivalis* on UC, which resulted in the exacerbation of inflammation in a mouse model. This exacerbation was due to PPAD, which induced an abnormal immune response and elevated the Th17/Treg ratio. Our findings offer further insights into the pathogenic effects of PPAD on systemic diseases.

## Results

### *P. gingivalis* exacerbated the intestinal inflammation of mice treated with DSS

**Fig. 1:**
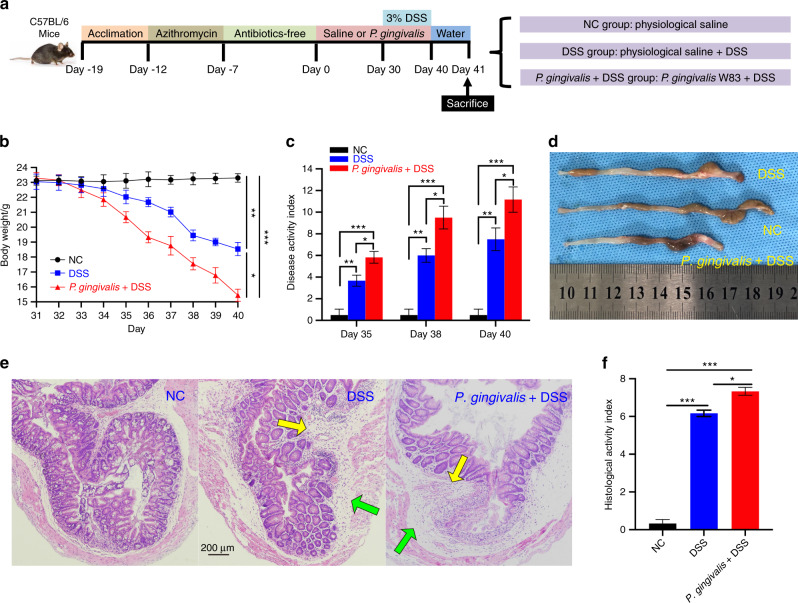
Clinical assessment indicates that *P. gingivalis* exacerbates UC. Schematic of the animal experimental design for the three groups (*n* = 6 in each group). **b** Mice with colitis showed significant body weight loss from 35th to 40th day after DSS induction. *P. gingivalis* suspension exacerbated body weight loss compared with DSS treatment (*P* < 0.01). **c** DAI scores calculated on 35th, 38th, and 40th day. The bar chart showing the typical presentation of inflammation in mice on 35th day after drinking the DSS solution. The DAI scores of the *P. gingivalis* + DSS group were significantly higher than those of the NC group and DSS group (*P* < 0.01). **d** The colons of the DSS group became shorter and exhibited more hyperemia and edema than those of the NC group (*P* < 0.01). The *P. gingivalis* + DSS group presented the shortest colons, the most severe inflammation, and the bloodiest stool (*P* < 0.05). **e** Representative H&E staining of colons from the three groups. The green and yellow arrows in the digital photographs of mice with colitis show the lymphocytic infiltration and partial exfoliation of the intestinal epithelium. The pathological changes observed after feeding a *P. gingivalis* suspension to mice with colitis by gavage were worse than those observed in the mice with colitis; these changes included large areas of deep ulcers, destruction of epithelial cells, exfoliation of intestinal epithelium and intestinal gland, exfoliation of basement membrane, and infiltration of neutrophils and lymphocytes. **f** The bar chart of the HAI scores indicated that damage in the *P. gingivalis* + DSS group was much worse than that in the DSS group (*P* < 0.01) and NC group (*P* < 0.01). The data are presented as the mean ± SD. *P < 0.05, **P < 0.01, ***P < 0.000 1 by independent 2-tailed Student’s *t* test and one-way ANOVA combined with Mann-Whitney U test

After 19 days of pretreatment, mice were inoculated with *P. gingivalis* strains on day 0 (Fig. 1a). In the first 30 days, no abnormalities were detected after each gavage of bacteria, and the body weight gain was consistent with the normal growth rate in all the mice. On the 32nd day, one day after the administration of DSS, the mice in the DSS groups exhibited the following changes: furs became dry, the luster disappeared, activity decreased, and water intake was reduced. On the 34th day, blood stains appeared in the perianal area. The appearance of occult blood and blood in the stool consistently increased, which was accompanied by body weight loss over time. As shown in Fig. 1b, the mice in the DSS group exhibited significantly greater body weight loss (*P* < 0.01) than the mice in the NC group, while the mice in the *P. gingivalis* + DSS group exhibited even greater weight loss (*P* < 0.05) than the mice in the DSS group.

In these experiments, the disease activity index (DAI) score increased with the time, indicating that the colitis model had been successfully established. The mice in the *P. gingivalis* + DSS group possessed higher DAI scores than those in the NC group (*P* < 0.000 1) and DSS group (*P* < 0.05) (Fig. 1c). Colon specimens of the mice in each group after 10 days of DSS exposure are presented in Fig. 1d and show that the colon length of the mice in the *P. gingivalis* + DSS group was shorter than that of the mice in the other two groups (NC group, *P* < 0.000 1; DSS group, *P* < 0.05), suggesting more severe inflammation.

The DSS-induced colitis model we used in the experiment was characterized by severely damaged intestinal epithelium. H&E colonic staining (Fig. 1e) showed that DSS-induced colitis caused multifocal small ulcers, disrupted epithelial cells and crypts, reduced goblet cell numbers and lymphocyte infiltration, and incomplete mucosal structure. However, the mice treated with both DSS and *P. gingivalis* exhibited exacerbated histological damage, as shown by the exfoliation of the intestinal epithelium and gland, fragmentation of the basement membrane, and serious infiltrations of the mucosa by inflammatory cells. The crypt defects were obvious, and diffuse mucosal hemorrhage and extended erosion were observed. The histological activity index (HAI) scores further indicated that the intestinal epithelium lesions of the *P. gingivalis* + DSS group were the worst among all the three groups (NC group, *P* < 0.000 1; DSS group, *P* < 0.05) (Fig. 1f).

### *P. gingivalis* increased Th17 cell number and decreased Treg numbers in colitis mice

**Fig. 2:**
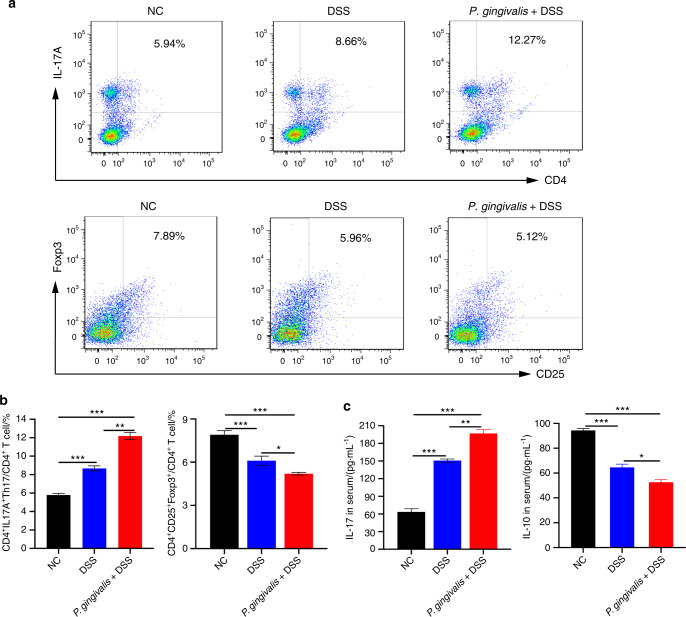
*P. gingivalis*-exacerbated UC is associated with an imbalance in Th17/Tregs. **a**, **b** Flow cytometric analysis and bar chart showing that compared with the NC group and the DSS group, *P. gingivalis* significantly induced the transformation of CD4^+^ T cells into proinflammatory Th17 cells (*P* < 0.01) and inhibited the generation of Tregs (*P* < 0.05). **c** ELISA showing that compared with the other two groups, *P. gingivalis* administration to mice with colitis significantly increased the expression level of IL-17 (*P* < 0.01) and decreased expression of IL-10 in serum (*P* < 0.05). All the indicators were measured in six replicates. The data are presented as the mean ± SD. *P < 0.05, **P < 0.01, ***P < 0.000 1 by independent 2-tailed Student’s *t* test and one-way ANOVA combined with Mann-Whitney U test

The ratio of Th17 cells to Tregs can reflect the systemic inflammatory balance in UC. Proinflammatory and anti-inflammatory cytokines play crucial roles in the progression of colitis by DSS-induced^43^. As shown in Fig. 2, we investigated the ratio of Th17 cells to Tregs in the spleen and the expression levels of the related cytokines in the serum to determine whether *P. gingivalis* could exacerbate colitis by altering the Th17/Treg balance.

Compared with the NC group, the DSS and *P. gingivalis* + DSS groups exhibited significantly increased numbers of CD4^+^ IL-17A^+^ cells (Th17 cell population) (*P* < 0.000 1) and protein expression level of IL-17 (*P* < 0.000 1) (Fig. 2a, b). In contrast, CD4^+^ CD25^+^ Foxp3^+^ cell numbers (Treg population) and IL-10 expression were significantly decreased in the DSS mice and were further decreased in the *P. gingivalis* + DSS mice (Tregs, *P* < 0.000 1; IL-10, *P* < 0.000 1) (Fig. 2c, d). Moreover, increases in the CD4^+^ IL-17A^+^ cell numbers and IL-17 expression and decreases in the CD4^+^ CD25^+^ Foxp3^+^ cell numbers and IL-10 expression were observed in the *P. gingivalis* + DSS groups compared to the DSS groups (Th17, *P* < 0.01; IL-17, *P* < 0.01; Tregs, *P* < 0.05; IL-10, *P* < 0.05).

### *P. gingivalis* and the PPAD gene complemented strain (comΔ*ppad*) increased Th17 cell numbers and decreased Treg cell numbers compared with the *P. gingivalis* strain in which the PPAD gene was deleted (Δ*ppad*)

**Fig. 3:**
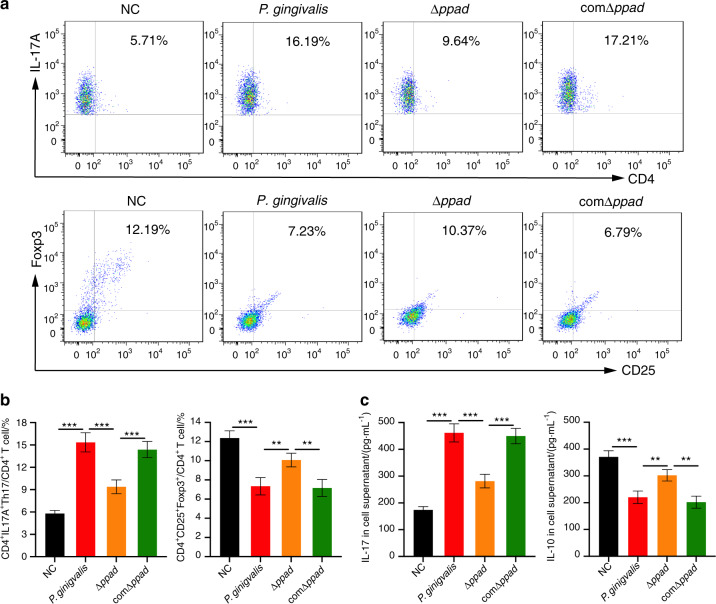
The proportions of T cell subsets and the expression of cytokines in vitro. **a**, **b** Flow cytometric analysis and bar chart showing that the *P. gingivalis*- and comΔ*ppad*-conditioned culture media significantly stimulated activated T lymphocytes to undergo a transformation into Th17 cells and decreased the generation of Tregs compared to Δ*ppad*-conditioned culture medium (*P* < 0.05). **c** ELISA showing that compared to Δ*ppad*-conditioned culture medium, *P. gingivalis*- and comΔ*ppad*-conditioned culture medium significantly increased the expression levels of IL-17 and decreased the expression levels of IL-10 in the cell supernatants (*P* < 0.05). Five replicates were analyzed per sample. The data are presented as the mean ± SD. *P < 0.05, **P < 0.01, ***P < 0.000 1 by independent 2-tailed Student’s *t* test and one-way ANOVA combined with Mann-Whitney U test

The identification results of the *P. gingivalis* W83 wild-type strain, Δ*ppad*, and comΔ*ppad* by PCR are shown in the Supplementary Figure. Lanes 1−3 show that the *P. gingivalis* 16S rRNA PCR products of the three strains are all positive, with about 200 bp. As shown in Lanes 4−6, the expressions of erythromycin resistance gene in *P. gingivalis* W83 wild-type strain is negative, while positive in Δ*ppad* and comΔ*ppad*. Lanes 7−9 demonstrate that the expressions of PPAD gene in *P. gingivalis* W83 wild-type strain and comΔ*ppad* are positive, but negative in Δ*ppad*. We used the supernatants of the cultures of the three strains as conditioned media to coculture T lymphocytes extracted from untreated C57BL/6 mice spleens. The proportion of IL-17A^+^ CD4^+^ cells and the protein expression level of IL-17A appeared to be significantly increased by *P. gingivalis*-conditioned growth medium (Th17, *P* < 0.000 1; IL-17, *P* < 0.000 1) and comΔ*ppad*-conditioned growth medium (Th17, *P* < 0.000 1; IL-17, *P* < 0.000 1) compared with Δ*ppad*-conditioned growth medium. In addition, the proportion of Tregs (*P. gingivalis*, *P* < 0.01; comΔ*ppad*, *P* < 0.01) and the expression level of IL-10 (*P. gingivalis*, *P* < 0.01; comΔ*ppad*, *P* < 0.01) were significantly decreased (Fig. 3).

### Deleting PPAD reduced the severity of UC

**Fig. 4:**
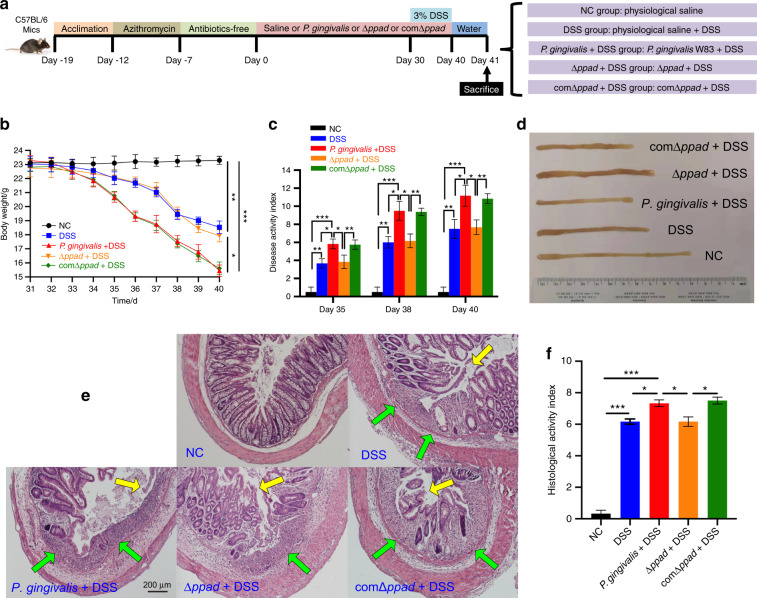
*P. gingivalis* and comΔ*ppad* exacerbate UC compared to Δ*ppad* in vivo. **a** Schematic of the animal experimental design in the five groups (*n* = 6 in each group). **b** The body weight loss of the DSS group and Δ*ppad* + DSS group was less than that of the *P. gingivalis* + DSS group and comΔ*ppad* + DSS group (*P* < 0.05). **c** The bar chart shows that the severity of colitis in the *P. gingivalis* + DSS group and comΔ*ppad* + DSS group was most serious among the five groups as determined assessed by the DAI scores (*P* < 0.05). The scores of the Δ*ppad* + DSS group were like those of the DSS group (*P* > 0.05). **d** Representative colons of the five groups. The specimens of the *P. gingivalis* + DSS group and comΔ*ppad* + DSS group were shorter than those in the other three groups (*P* < 0.05). The Δ*ppad* + DSS group presented a length that was approximately similar to that of the DSS group (*P* > 0.05). **e** Representative H&E staining of the colons from the five groups. The comΔ*ppad* + DSS group showed pathological lesions similar to those of the *P. gingivalis* + DSS group, with large areas of deep ulcers, destruction of epithelial cells, exfoliation of intestinal epithelium, and intestinal gland, fragmentation of basement membrane, and infiltration of neutrophils and lymphocytes. Δ*ppad* administration to mice with colitis led to pathological changes similar to those of mice with colitis, including lymphocytic infiltration and partial exfoliation of the intestinal epithelium. **f** Bar chart showing that the HAI score of the *P. gingivalis* + DSS group was equal to that of the comΔ*ppad* + DSS group (*P* > 0.05) and worse than that of the Δ*ppad* + DSS group and DSS group (*P* < 0.05). The data are presented as the mean ± SD. *P < 0.05, **P < 0.01, ***P < 0.000 1 by independent 2-tailed Student’s *t* test and one-way ANOVA combined with Mann-Whitney U test

Compared with the DSS group, the clinical indicators of the Δ*ppad* + DSS group revealed that Δ*ppad* did not exacerbate the colitis phenotype (body weight loss, *P* > 0.05; DAI, *P* > 0.05; colon length, *P* > 0.05) (Fig. 4b–d). The mice in the Δ*ppad* + DSS group exhibited histopathological characteristics similar to those in the DSS group (HAI, *P* > 0.05) (Fig. 4e, f). The intestinal inflammation of the Δ*ppad* + DSS group was significantly milder than that of the *P. gingivalis* + DSS group (body weight loss, *P* < 0.05; DAI, *P* < 0.05; colon length, *P* < 0.05; HAI, *P* < 0.05) and comΔ*ppad* + DSS group (body weight loss, *P* < 0.05; DAI, *P* < 0.05; colon length, *P* < 0.05; HAI, *P* < 0.05) (Fig. 4b–f).

**Fig. 5:**
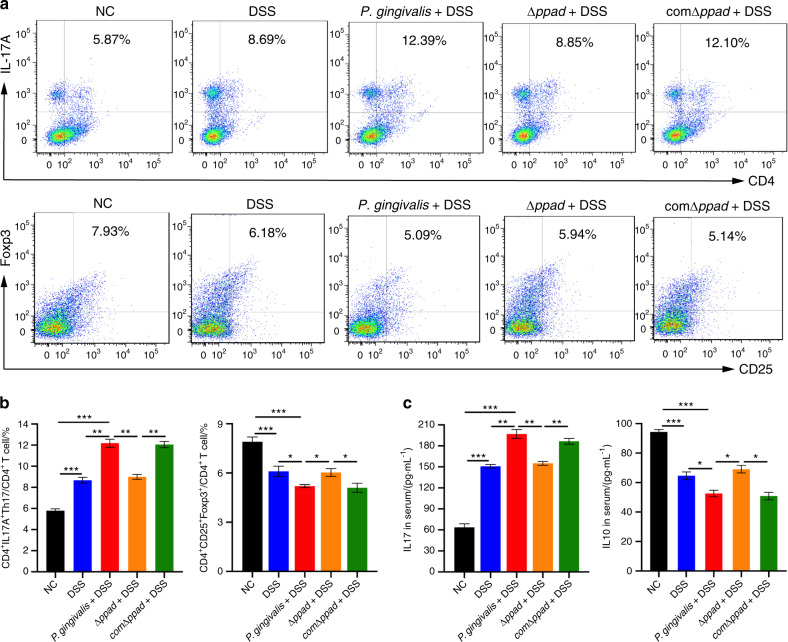
*P. gingivalis*- and comΔ*ppad*-exacerbated UC is associated with an imbalance in Th17/Tregs. **a**, **b** Flow cytometric analysis and bar chart showing that compared with Δ*ppad*, *P. gingivalis* and comΔ*ppad* induced the transformation of CD4^+^ T cells into proinflammatory Th17 cells and simultaneously inhibited the generation of Tregs (*P* < 0.05), but no difference was observed between mice administered Δ*ppad* and DSS and mice with colitis administered physiological saline (*P* > 0.05). **c** ELISA showing that compared with Δ*ppad*, *P. gingivalis* and comΔ*ppad* administration caused the increased expression of IL-17 and decreased expression of IL-10 in serum (*P* < 0.05). The expression levels of the two cytokines were similar in the mice administered Δ*ppad* and DSS mice and the mice with colitis (*P* > 0.05). All indicators were measured with six replicates. The data are presented as the mean ± SD. *P < 0.05, **P < 0.01, ***P < 0.000 1 by independent 2-tailed Student’s *t* test and one-way ANOVA combined with Mann-Whitney U test

As shown in Fig. 5, the flow cytometry and ELISA results demonstrated that the administration of Δ*ppad* by gavage to mice with colitis mice did not promote abnormal immune responses of splenic T lymphocytes. The proportions of T cell subsets and the expression levels of cytokines were similar between the Δ*ppad* + DSS group and DSS group (Th17, *P* > 0.05; IL-17, *P* > 0.05; Tregs, *P* > 0.05; IL-10, *P* > 0.05). Compared with the Δ*ppad* + DSS groups, the *P. gingivalis* + DSS group and comΔ*ppad* + DSS group exhibited significantly increased percentages of Th17 cells (*P. gingivalis*, *P* < 0.01; comΔ*ppad*, *P* < 0.01) (Fig. 5a, b) and the expression levels of proinflammatory cytokine of IL-17 (*P. gingivalis*, *P* < 0.01; comΔ*ppad*, *P* < 0.01) (Fig. 5c), and decreased percentages of the suppressor T cells of Tregs (*P. gingivalis*, *P* < 0.05; comΔ*ppad*, *P* < 0.05) (Fig. 5a, b) and expression levels of IL-10 (*P. gingivalis*, *P* < 0.05; comΔ*ppad*, *P* < 0.05) (Fig. 5c).

### PPAD induced an abnormal proinflammatory immune response in the colon

**Fig. 6:**
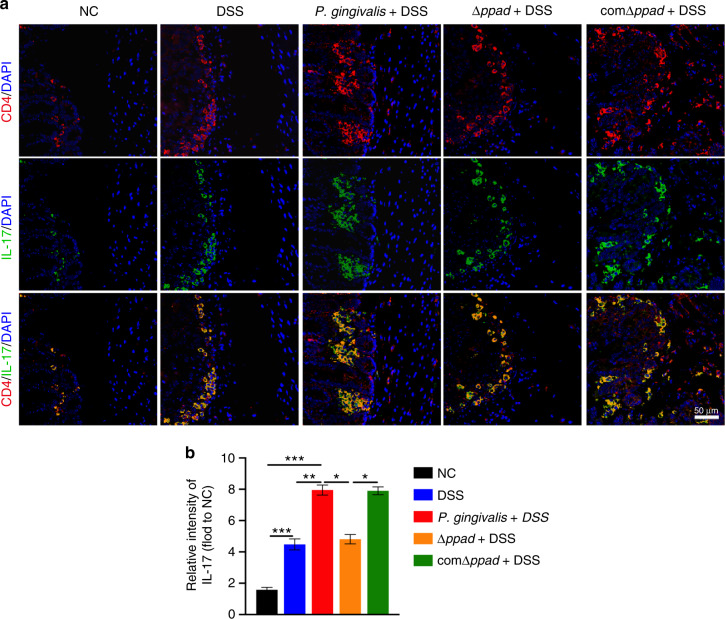
*P. gingivalis* and comΔ*ppad* exacerbate UC by upregulating IL-17 compared to Δ*ppad* as shown by immunofluorescence staining analysis. **a** Colon sections of mice from the NC group, DSS group, *P. gingivalis* + DSS group, Δ*ppad* + DSS group, and comΔ*ppad* + DSS group were stained with anti-CD4 and anti-IL-17 antibodies. Scale bar: 50 μm. **b** The IL-17 intensity in the CD4^+^ T cells of mice was quantified (*n* = 6 in each group). The data are presented as the mean ± SD. *P < 0.05, **P < 0.01, ***P < 0.000 1 by independent 2-tailed Student’s *t* test and one-way ANOVA combined with Mann-Whitney U test

We harvested mouse colons at the end of the experiment on the 41st day. Immunostaining indicated a dramatic increase in the expression of IL-17 and a decrease in the expression of IL-10 in the colons of the *P. gingivalis* + DSS group and comΔ*ppad* + DSS group compared with those of the control group (IL-17, *P* < 0.000 1; IL-10, *P* < 0.000 1). The expression of IL-17 was significantly increased and the expression of IL-10 was decreased in the CD4^+^ T cells of the colon samples of the *P. gingivalis* + DSS group and comΔ*ppad* + DSS group compared with those of the DSS group and Δ*ppad* + DSS group (IL-17, *P* < 0.05; IL-10, *P* < 0.05) (Fig. 6), indicating that bacteria that secrete PPAD can cause proinflammatory immune responses of CD4^+^ T cells in colon tissue.

## Discussion

Periodontitis and UC are both chronic inflammatory diseases mediated by complex interactions between immune responses and the microbes. *P. gingivalis*, as the major pathogenic bacterium that causes periodontitis, is considered a risk factor for many systemic diseases, such as diabetes mellitus, rheumatoid arthritis, lupus erythematosus, Alzheimer’s disease, and UC^44^. In our in vivo study, the mice treated with *P. gingivalis* by gavage exhibited a more severe clinical presentation than the mice treated with DSS alone, indicating that *P. gingivalis* can exacerbate the inflammation of UC. Kitamoto et al. found that symbiotic oral pathogens and effector memory T cells did not produce IFN-γ during periodontitis^8^. However, after reaching the intestine, these bacteria-cell symbionts produced IFN-γ and caused colitis. Intestinal inflammation disrupts the homeostasis of normal intestinal flora, allowing the oral bacteria to defeat and displace the resident bacteria in the intestine. Furthermore, oral inflammation, such as periodontitis, can increase the number of oral pathogens. After reaching a certain threshold, this number will increase the probability of the successful passage of these oral pathogens through the acidic environment of the stomach. The synergistic effect of these two conditions can promote the ectopic oral bacterial colonization of the intestine. In this regard, oral bacteria will exacerbate existing intestinal inflammation, but do not affect healthy intestines^8^.

Despite the fact that inflammation is a distinguishing characteristic of periodontitis, *P. gingivalis* not only causes inflammation but also acts as a potent inducer of abnormal immune responses^45^. *P. gingivalis* can induce abnormal cellular immunological responses^46,47^. In patients with periodontitis, Th17 cell numbers and related cytokine production are increased and positively correlated with periodontal destruction due to *P. gingivalis*, whereas Treg cell numbers and related cytokine production are decreased and negatively correlated with periodontal destruction^22^. The imbalance of Th17/Tregs has recently been proven to be a cause of the pathogenesis of periodontitis and many autoimmune diseases^48,49^. An emerging viewpoint is that UC is associated with immune-inflammatory responses, which are the result of the abnormal immune responses of T lymphocytes to specific microbes in genetically susceptible populations^50^. We also investigated the proportions of Th17 and Tregs among splenic T lymphocytes and the expression levels of IL-17 and IL-10 in the serum. According to the results, we concluded that *P. gingivalis* significantly increased the proportion of Th17 cells and the level of IL-17 and inversely decreased the proportion of Tregs and the level of IL-10. Bacteria-reactive CD4^+^ T cells in the gut have been proposed to play an influential role in the development of IBD^51,52^. Under certain circumstances, Th17 cells in the intestine undergo pathogenic transformation to produce IFN-γ and cause intestinal inflammation^53,54^. These cells can also trigger many autoimmune diseases, such as UC, by enhancing the permeability of cells and promoting the recruitment and activation of inflammatory cells, which consequently leads to inflammatory changes in the body^55^. A number of studies have discovered that the number of Th17 cells in the peripheral blood of UC patients is significantly increased and positively correlated with disease activity^56,57^. Li et al^58^. further confirmed that the inhibition of Th17 cell function in intestinal tissues significantly reduces the degree of colitis in mice with DSS-induced. High levels of IL-17 in the peripheral blood and colonic mucosa can lead to a highly inflammatory state in the host, which is closely related to the pathogenesis of UC^59^.

Tregs are a subset of T cells that are identified by CD4 and CD25 expression, are mainly responsible for the negative regulation of the inflammatory responses, and perform the functions of preventing, weakening, or terminating various inflammatory reactions^21^. Tregs can inhibit the activities of dendritic cells and macrophages that contribute to innate immunity^60^. Tregs also inhibit the development of various inflammatory diseases by secreting the critical cytokine of IL-10, which is characterized by its anti-inflammatory properties. IL-10 can downregulate the transcription and secretion of IL-6, TNF-α, and some other proinflammatory factors in the intestinal tract to suppress T cells and maintain tolerance functions, and maintaining mucosal homeostasis^61^. The metabolites of intestinal microorganisms can alleviate intestinal inflammatory symptoms in mice by increasing the number of Tregs and the expression level of IL-10 ^62^. It has been noted that the balance of Th17 cells and Tregs is very important for controlling inflammation and maintaining intestinal immune homeostasis^63^.

**Fig. 7:**
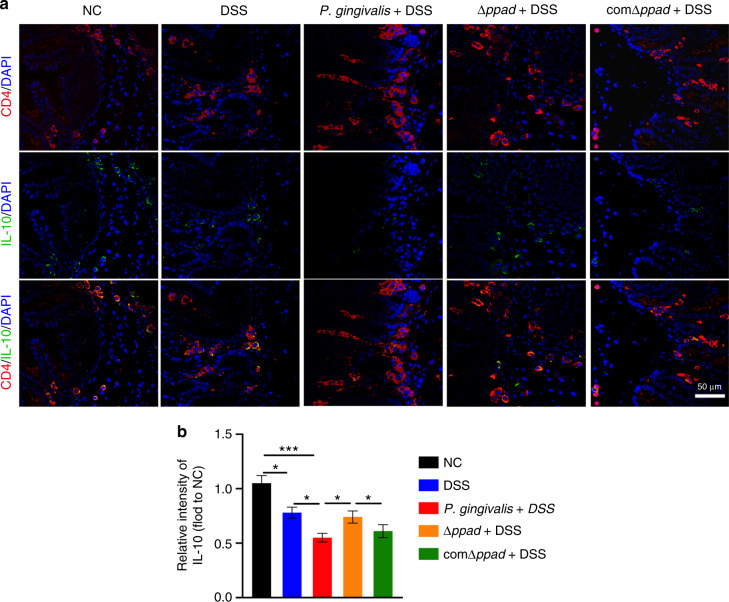
*P. gingivalis* and comΔ*ppad* exacerbate UC by downregulating IL-10 compared to Δ*ppad* as shown by immunofluorescence staining analysis. **a** Colon sections of mice from NC group, DSS group, *P. gingivalis* + DSS group, Δ*ppad* + DSS group, and comΔ*ppad* + DSS group were stained with anti-CD4 and anti-IL-10 antibodies. Scale bar: 50 μm. **b** The IL-10 intensity in the CD4^+^ T cells of mice was quantified (*n* = 6 in each group). The data are presented as the mean ± SD. *P < 0.05, **P < 0.01, ***P < 0.000 1 by independent 2-tailed Student’s *t* test and one-way ANOVA combined with Mann-Whitney U test

*P. gingivalis* expresses dozens of virulence factors, such as gingipains, fimbrillin peptides, lipopolysaccharides, outer membrane vesicles, and various enzymes. By effectively manipulating the immunosuppression through the various virulence factors, *P. gingivalis* may play a role in periodontitis and related systemic diseases. Much of the pathogenicity of *P. gingivalis* is owing to its ability to damage the host’s immune defenses. PPAD is an enzyme secreted by *P. gingivalis* in the oral cavity and can induce the abnormal inflammatory responses and autoimmune reactions in the host by increasing protein citrullination^35,36,64^. To confirm the proinflammatory effect of PPAD, we constructed Δ*ppad* by silencing the expression function of PPAD, and we restored the PPAD gene by homologous recombination to construct comΔ*ppad*. Murine splenic T lymphocytes were stimulated with *P. gingivalis*-, Δ*ppad*-, and comΔ*ppad*-conditioned media. It is clear that *P. gingivalis* and comΔ*ppad* can significantly promote the transformation of CD4^+^ T cells into proinflammatory Th17 cells while inhibiting the generation of Tregs. By investigating cocultured cell supernatants, it was observed that *P. gingivalis* and comΔ*ppad* induced significant increases in the expression of IL-17 and decreases in the expression of IL-10. In the last part of this study, we verified the impact of the PPAD gene by orally gavaging live *P. gingivalis*, Δ*ppad*, and comΔ*ppad* separately into UC mice. The oral gavage of *P. gingivalis* and comΔ*ppad* to UC mice resulted in more severe clinical symptoms and colonic damage than the oral gavage of Δ*ppad* and the control to UC mice. The immunological indicator results were similar to the results of the in vitro cytological experiments. Compared with Δ*ppad* and the control, wild-type *P. gingivalis* strain and the complemented strain stimulated increased proportions of Th17 cells and increased expression of IL-17 but decreased proportions of Tregs and expression of IL-10 in mice with UC. PAD is activated extracellularly or in the cytoplasm by calcium ions, and activated PAD can promote protein citrullination, disrupt the host immune balance, and cause excessive inflammatory responses^65^. Citrulline levels are biomarkers of human intestinal function. Citrullination is a posttranslational modification that occurs by the conversion of arginine residues, and it can lead to pathogenesis by regulating the transcription of cytokines and the production of proinflammatory proteins^66^. PAD-mediated hypercitrullination is able to inhibit the expression of Th2 cytokines and amplifying the production of Th17 cytokines, resulting in an imbalance in the Th17/Tregs ratio^67^. Our immunofluorescence staining results showed that the wild-type strain expressing PPAD caused an increased expression of the proinflammatory cytokine of IL-17 and decreased expression of the protective cytokine of IL-10 in colon tissues. The same result was also observed with the complemented strain. These results indicate that the PPAD gene can result in an abnormal immune response in the intestinal tissue Fig. 7.

In this study, we can conclude that *P. gingivalis* exacerbates intestinal inflammation in UC via PPAD. The possible mechanism is that PPAD induces the transformation of naive CD4^+^ T cells into proinflammatory Th17 cells and inhibits the generation of anti-inflammatory Tregs. The interaction between PPAD and citrullinated proteins and the mechanism by which colitis is exacerbated is still unclear. We will explore the mechanism by which PPAD exacerbates UC in future studies. The in-depth study of PPAD has broadened our horizon and provides a new research direction for exploring the related virulence factors expressed by periodontal pathogens and the abnormal immune responses of intestinal diseases. In the future, we can target PPAD to prevent the progression and exacerbation of UC by increasing the quantity and function of Tregs and maintaining the balance of Th17/Tregs in intestinal homeostasis.

## Materials and methods

### Preparation of bacterial strains and growth conditions

The *P. gingivalis* W83 wild-type strain was cultured for 5 days at 37 °C in Brain Heart Infusion (BHI) broth supplemented with vitamin K (1 μg·mL^−1^), hemin (5 μg·mL^−1^) and 5% defibrinated sheep blood in an anaerobic chamber as previously described^68^. The *Escherichia coli* (*E. coli*) DH5α strain was used for DNA cloning and cultured in a broth and on agar plates containing the appropriate antibiotics.

### Construction and identification of *P. gingivalis* mutant strain (Δ*ppad*) and complemented strain (comΔ*ppad*)

The PPAD gene is an outer membrane protein, which is located at the position of 1509227–1510897 bp (GenBank accession number 2552184; locus tag PG1424) in the genome of the *P. gingivalis* W83 strain, and the total length of the gene is 1671 bp. The upstream region of the PPAD gene and the erythromycin resistance cassette ermF/ermAM were amplified by PCR and inserted into the XbaI and SphI sites and the SmaI and XbaI sites, respectively, of the pUC19 plasmid (Genscript Biotech Co., Nanjing, China). Then, the downstream region of the PPAD gene was inserted into the SacI and SmaI sites to construct the deletional inactivation plasmid. Alternatively, instead of the downstream region of the PPAD gene was inserted into the SacI and SmaI sites to construct the complemented plasmid. The correct placement and orientation of the inserted DNA segments were verified by sequencing. These two modified plasmids were integrated into the *P. gingivalis* W83 genome by electroporation to generate Δ*ppad* and comΔ*ppad*. The Δ*ppad* and comΔ*ppad* were subcultured on erythromycin-containing selection plates^3^.

To identify *P. gingivalis* W83, Δ*ppad* and comΔ*ppad*, total DNA of these three strains were extracted by Genomic DNA Mini Preparation Kit (#D0063, Beyotime Biotechnology, Shanghai, China), PCR was performed with primers targeting *P. gingivalis* 16S rRNA-, Erm-, or PPAD-specific regions, which were designed by Primer Premier 6.0 (supplementary table). The three strains were centrifuged at 10 000 × *g* for 10 min and then adjusted to a final concentration of 1 × 10^8^ colony forming units (CFUs)/mL based on ultraviolet spectrophotometry colorimetry at 450 nm for further use.

### Animals and dextran sodium sulfate (DSS)-induced UC

Six-week-old, healthy, specific pathogen-free (SPF) female C57BL/6 mice weighing approximately (20.0 ± 1.0) g were obtained from Beijing Vital River Laboratory Animal Technology Co. We used the female sex only, as female C57BL/6 mice have been reported to be more susceptible to UC modeling in the literature^69^. All the mice were acclimatized under SPF conditions, exposed to 12 h cycles of light and dark, and fed with standard rodent chow and water ad libitum in clear cages for one week. Then, all the mice were administered azithromycin in their drinking water (10 mg per 500 mL) for 5 days to eliminate the original intestinal flora, followed by a 7-day antibiotic-free period before the initiation of DSS administration at eleven weeks of age^70^. DSS (#160110, molecular weight: 36 000–50 000; MP Biomedicals, Santa Ana, USA) dissolved in drinking water (3% (wt/vol)) was provided ad libitum to those mice for 10 days to induce an acute colitis^71^.

### Grouping and experimental design

All the mice were randomly divided into five groups (*n* = 6 per group): the normal control group (NC), DSS group (DSS), *P. gingivalis* + DSS group (*P. gingivalis* + DSS), Δ*ppad* + DSS group (Δ*ppad* + DSS), and comΔ*ppad* + DSS group (comΔ*ppad* + DSS). The mice in the *P. gingivalis* + DSS group, Δ*ppad* + DSS group, and comΔ*ppad* + DSS group were administered a total of 10^8^ CFUs of live *P. gingivalis* W83, Δ*ppad*, or comΔ*ppad* suspended in 100 μL sterile saline twice per week by oral gavage for the entire 40 days (days 0−40) and an additional 3% DSS for the last 10 days (days 31–40)^71–73^. In the DSS groups, the mice were orally gavaged with saline twice per week (days 0−40) and 3% DSS for 10 days (days 31–40). The mice in the NC group were orally gavaged with saline twice per week for the entire 40 days and offered normal drinking water.

The daily observation was conducted to record the weight, water/food consumption, piloerection, stool consistency, occult blood, and hematochezia throughout the experiment. At the end of the experiment (day 41, Fig. 1a), peripheral blood was collected from the inner canthus for cytokine detection. All the mice were humanely sacrificed, and colon segments and spleens were harvested for follow-up analyses.

### Assessment of the severity of colitis by measuring the DAI and HAI

In the colitis model, disease severity is usually related to shortened colon length caused by intestinal inflammation^74^. Oral administration of DSS leads to loss of body weight, changes in stool consistency, and bloody diarrhea that mimic the parameters of the clinical presentation of humans with UC and can be used to evaluate the DAI score^71^. Colon segments were harvested, fixed in 4% paraformaldehyde, dehydrated with ethanol, and embedded in paraffin. Paraffin sections were deparaffinized, rehydrated, and stained with hematoxylin and eosin (H&E) for histological analyses and calculating the HAI score^75^ (supplementary table).

### Culture of T cells with *P. gingivalis*-, Δ*ppad*-, or comΔ*ppad*-conditioned medium

*P. gingivalis*, Δ*ppad*, and comΔ*ppad* were maintained in BHI broth at 37 °C for 24 h in an anaerobic chamber. A total of 1 × 10^8^ CFU bacteria were quantified, and the supernatants of three strains were collected by centrifugation at 10 000 × *g* and filtered through a 0.22 μm filter. Twelve-well plates were coated with anti-mouse CD3 (2 µg·mL^−1^ in PBS, 1 mL per well) (#100340, Biolegend, California, USA) per well at 4 °C overnight before T cell isolation.

Spleens were collected from healthy C57BL/6 mice and passed through a 70 μm cell strainer. Further clearing of the noncellular debris was achieved by centrifugation at 500 × *g* for 5 min. The resulting debris-free single-cell suspension was washed and filtered through a 40 μm cell strainer and centrifuged again. After the final resuspension, the total numbers of T lymphocytes were counted under a hemocytometer. The T lymphocytes were washed and resuspended in RPMI-1640 medium (#21870084, Gibco, Carlsbad, USA) supplemented with 10% fetal bovine serum (#10100139C, Gibco, Carlsbad, USA), penicillin-streptomycin solution (100 U·mL^−1^ penicillin/100 μg·mL^−1^ streptomycin) (#SV30010, HyClone, South Logan, USA), anti-mouse CD28 antibody (2 μg·mL^−1^) (#102116, Biolegend, California, USA), and TGF-β (2 ng·mL^−1^) (#100-21-2, Peprotech, Rocky Hill, USA). IL-6 (50 ng·mL^−1^) (#216-16-2, Peprotech, Rocky Hill, USA) was added to the RPMI-1640 medium to differentiate Th17 cells, while IL-2 (2 ng·mL^−1^) (#212-12-5, Peprotech, Rocky Hill, USA) was added to the medium to differentiate Tregs. T lymphocytes were cocultured with the bacterial medium in 12-well plates without the coating buffer in humidified air with 5% CO_2_ at 37°C for 3 days. Then, the cells and supernatants were harvested for analyses.

### Determination of the percentages of T cell subsets by flow cytometric analysis

A total of 1 × 10^6^ splenic T lymphocytes in vivo and three kinds of in vitro cocultured T cells described above were separately washed and resuspended in FACS buffer containing phosphate-buffered saline (PBS) and 0.5% bovine serum albumin (BSA). The suspension was incubated with fluorescently labeled monoclonal antibodies for 1 h at 4 °C in the dark. Anti-mouse PerCP-CD4 (#46-0041-82, Thermo Fisher Scientific, Waltham, USA) and anti-mouse PE-IL-17 (#12-7177-81, Thermo Fisher Scientific, Waltham, USA) monoclonal antibodies were prepared to identify Th17 cells, while anti-mouse PerCP-CD4, anti-mouse APC-CD25 (#17-0251-82, Thermo Fisher Scientific, Waltham, USA), and anti-mouse PE-Foxp3 (#12-5773-82, Thermo Fisher Scientific, Waltham, USA) monoclonal antibodies were prepared to identify Tregs.

After washing three times, the cells were resuspended again in PBS containing 0.1% BSA for flow cytometry (#Canto II, BD Biosciences, New Jersey, US). The data were analyzed with FlowJo (#V10.5, BD Biosciences, New Jersey, US).

### Detection of the cytokines IL-17 and IL-10 in sera and cell supernatants

Peripheral blood was harvested from the inner canthus and then centrifuged at 3,000 rpm for 10 min to isolate the serum. Mouse cytokine ELISA kits (anti-mouse IL-17A: #88-7371-88, Thermo Fisher Scientific, Waltham, USA; anti-mouse IL-10: #88-7105-88, Thermo Fisher Scientific, Waltham, USA) were used to analyze the levels of the cytokines IL-17 and IL-10 in the sera and cell supernatants. The samples were analyzed in triplicate, and the concentrations were calculated according to standard curves.

### Immunofluorescence staining analysis

Paraffin sections (6 μm) were deparaffinized, rehydrated, prepared, and double-stained with the following antibodies: (i) rat anti-CD4 (1:500; BD Pharmingen, California, USA) with rabbit anti-IL-17 (1:500; Novus Biologicals, Littleton, Colorado, USA) and (ii) rat anti-CD4 (1:500; BD Pharmingen, California, USA) with goat anti IL-10 (1:200; R&D, California, USA). The sections were incubated with the antibodies in a humidified chamber overnight at 4 °C. After being washed with PBS, the sections were incubated with a mixture of Alexa 488-conjugated donkey anti-goat (1:500; Jackson ImmunoResearch, Pennsylvania, USA) and Cy3-conjugated donkey anti-rat (1:500; Jackson ImmunoResearch, Pennsylvania, USA) secondary antibodies for 2 h at room temperature. All of the sections were counterstained with DAPI (1:10 000; #PP0131, Beyotime Biotechnology, Shanghai, China). Images were obtained using a FluoView confocal microscope (#FV3000, Olympus, Tokyo, Japan). Immunostaining quantification was completed using ImageJ software.

### Statistical analysis

GraphPad Prism 8.0 (GraphPad software Corp., California, USA) was used for the statistical analysis. Comparisons between two groups were performed using an independent two-tailed Student’s *t* test, and one-way analysis of variance (ANOVA) combined with the Mann−Whitney U test was used to determine the significance of the differences among multiple groups. The data are shown as the means ± SDs. *P* < 0.05 was considered statistically significant.

## Supplementary information

Supplementary figure legend and table

Electropherogram of identification of the *P. gingivalis* W83 wild-type strain, Δ*ppad*, and comΔ*ppad*
