# Supplementary material for: Porphyromonas gingivalis exacerbates ulcerative colitis via Porphyromonas gingivalis peptidylarginine deiminase
Source: Int J Oral Sci. 2021 Sep 30;13:31. doi: 10.1038/s41368-021-00136-2 (PMC8484350; doi:10.1038/s41368-021-00136-2)
Supplement: Supplementary file 1 — Supplementary figure legend and table [file 41368_2021_136_MOESM1_ESM.docx]

**SUPPLEMENTARY FIGURE LEGEND**

**Electropherogram of identification of the *P. gingivalis* W83 wild-type strain, Δ*ppad*, and comΔ*ppad*.** M: 1kb DNA Marker. Lane 1, 2, and 3: the PCR products of the *P. gingivalis* 16S rRNA (1：the *P. gingivalis* W83 wild-type strain, 2：Δ*ppad*, 3：comΔ*ppad*). Lane 4, 5, and 6: the PCR products of internal primer of erythromycin resistance gene (4：the *P. gingivalis* W83 wild-type strain, 5：Δ*ppad*, 6：comΔ*ppad*). Lane 7, 8, and 9: the PCR products of internal primer of PPAD gene (7：the *P. gingivalis* W83 wild-type strain, 8：Δ*ppad*, 9：comΔ*ppad*).

**SUPPLEMENTARY TABLE**

**Table 1. Primers used in this study**

| Description | Sequence (5’ to 3’) |
| --- | --- |
| *P. gingivalis* W83 | F: TGTAGATGACTGATGGTGAAAACC |
|  | R: ACGTCATCCACACCTTCCTC |
| Δ*ppad* upstream region | F: GCTCTAGATGGAATCCGTGAGACAATG |
|  | R: TAAGCATGCGATATTTGTCGGAAGGACTC |
| ermF/ermAM | F: TATTAGGCCTATAGCTTCCGCTATT |
|  | R: AATAGGCCTTAGTAACGTGTAACTTT |
| Δ*ppad* downstream region | F: AAGAGCTCAAGCACGTAATAAGGACAATGA |
|  | R: TTATCCCGGGTGTTCCTGAACATATGATAAGATCT |
| comΔ*ppad* | F: AAGAGCTCAAGCACGTAATAAGGACAATGA |
|  | R: TTATCCCGGGTGTCTACCTGAGGAGTATTCT |
| PPAD | F: TTATTTGAGAATTTTCATTGTCTCA |
|  | R: ATGAAAAAGCTTTTACAGGCTAAA |

**Table 2. Disease activity index grading**

| Feature graded | Grade | Description |
| --- | --- | --- |
| Weight loss | 0 | None |
|  | 1 | 1-5% |
|  | 2 | 5-10% |
|  | 3 | 10-15% |
|  | 4 | ＞15% |
| Diarrhea | 0 | Normal |
|  | 2 | Loose stools |
|  | 4 | Watery diarrhea |
| Bleeding | 0 | No bleeding |
|  | 2 | Slight bleeding |
|  | 4 | Gross bleeding |

**Table 3. Histological activity index grading**

| Feature graded | Grade | Description |
| --- | --- | --- |
| Epithelium | 0 | Normal morphology |
|  | 1 | Minimal loss of goblet cells |
|  | 2 | Extensive loss of goblet cells |
|  | 3 | Loss of crypts |
|  | 4 | Loss of crypts in large areas |
| Infiltration | 0 | No infiltrate |
|  | 1 | Infiltrate around the crypt basis |
|  | 2 | Infiltrate in the muscularis mucosa |
|  | 3 | Extensive infiltrate in the muscularis mucosa with edema |
|  | 4 | Infiltration in the submucosa |
