# Supplementary figures and images for: Porphyromonas gingivalis exacerbates ulcerative colitis via Porphyromonas gingivalis peptidylarginine deiminase
Source: Int J Oral Sci. 2021 Sep 30;13:31. doi: 10.1038/s41368-021-00136-2 (PMC8484350; doi:10.1038/s41368-021-00136-2)

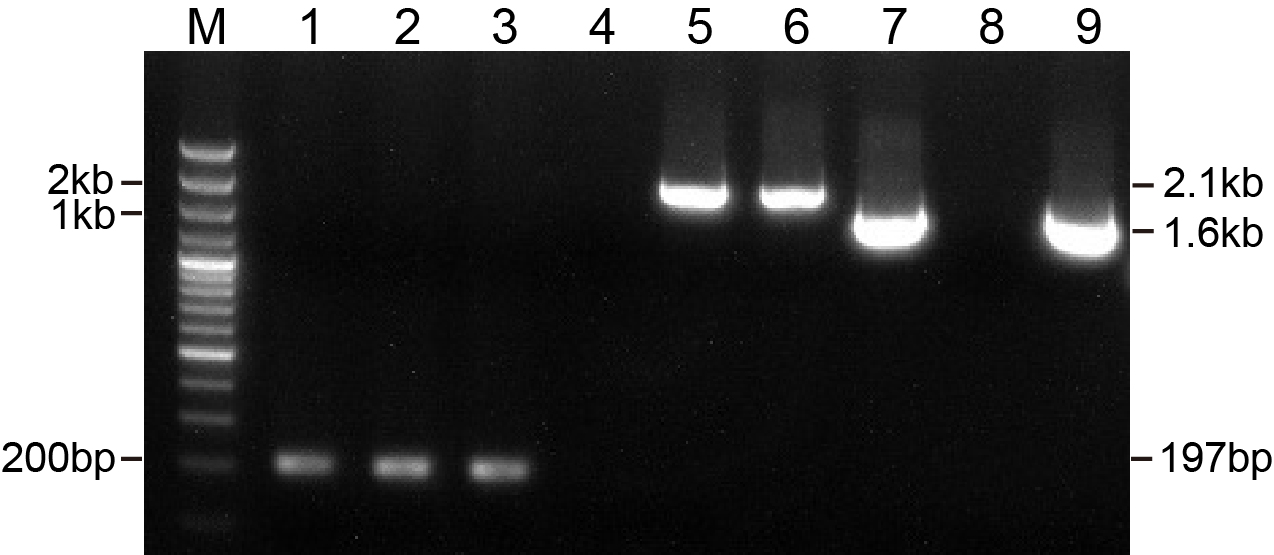

Supplement: Supplementary file 2 — Electropherogram of identification of the P. gingivalis W83 wild-type strain, Δppad, and comΔppad [file 41368_2021_136_MOESM2_ESM.jpg]
